# Exploring the inclusion of under-served groups in trials methodology research: an example from ethnic minority populations’ views on deferred consent

**DOI:** 10.1186/s13063-021-05568-z

**Published:** 2021-09-03

**Authors:** Timia Raven-Gregg, Victoria Shepherd

**Affiliations:** 1 School of Medicine, Cardiff University, Cardiff, UK; 2 Centre for Trials Research, Cardiff University, 4th Floor, Neuadd Meirionnydd, Heath Park, Cardiff, CF14 4YS UK

**Keywords:** Deferred consent, Under-served, Inclusivity, Patient views, Ethnic minority, Clinical trial

## Abstract

**Background:**

Deferred consent is used to recruit patients in emergency research, when informed consent cannot be obtained prior to enrolment. This model of consent allows studies to recruit larger numbers of participants, especially where a surrogate-decision maker may be unavailable to provide consent. Whilst deferred consent offers the potential to promote trial diversity by including under-served groups, it is ethically complex and views about its use amongst these populations require further exploration. The aim of this article is to build upon recent initiatives to improve inclusivity in trials, such as the NIHR INCLUDE project, and consider whether trials methodology research is inclusive, focusing on ethnic minority populations’ attitudes towards the use of deferred consent.

**Main text:**

Findings from the literature suggest that research regarding attitudes toward recruitment methods like deferred consent largely fail to adequately represent ethnic minorities. Many studies fail to report the composition of patient samples or conduct analyses on any differences between specific patient groups. In those that do, the categorisation of ethnic groups is ambiguous. Frequently diversely different groups are considered as more homogenous than they are. Whilst deferred consent is deemed generally acceptable, analysis of patient sub-groups shows that this attitude is not universal. Those from racial and ethnic minority backgrounds reported higher levels of unacceptability, which was impacted by previous first or second-hand experience of its use and historical mistrust in research. However, whilst deferred consent was found to increase the numbers of black participants enrolled in some trials, their over-enrolment in other trials may raise further concerns.

**Conclusions:**

Inclusivity in clinical trials is important, as highlighted by the COVID-19 pandemic. To improve this, we must ensure that methodological studies such as those exploring attitudes to research are inclusive. More effort is needed to understand the views of under-served groups, such as ethnic minorities, toward research in order to improve participation in clinical trials. Our findings echo those from the INCLUDE project, in that better reporting is needed and increasing the confidence of ethnic minority groups in research requires improving representation throughout the research process. This will involve diversifying research teams and ethics committees.

## Background

Deferred consent is a strategy used in emergency clinical research, whereby participants are enrolled and included in research without prospectively providing consent. Typically, this method is used when the intervention is required urgently but the patient lacks the capacity to consent to research and there is no time to involve a surrogate-decision maker to consent on their behalf. This includes in prehospital ambulance research [1], in critical care settings [2], and in a number of high-profile COVID-19 studies [3]. In such circumstances, if capacity and an ability to understand the study information is regained during the study, patients are then informed and consent is sought for continued participation or withdrawal from the study, or a surrogate is approached on their behalf [4]. As randomisation, delivery of intervention, and data collection will usually have already occurred at this point, this consent is generally to continue data collection. This approach is in keeping with the Medicines for Human Use Clinical Trials Regulations (2006) for clinical trials of medicines in the United Kingdom (UK) and the Mental Capacity Act (2005) for other types of research in England and Wales. Additionally in the UK, where a family member is unavailable to act as surrogate, someone acting in a professional capacity, such as member of their medical team, may take on this role provided they are not involved in the study. However, international differences exist between countries’ approaches to the use of deferred consent in practice. Despite European Union (EU) regulations allowing emergency research using deferred consent for incapacitated patients, differences remain between the use of deferred consent amongst EU member states [5]. Additionally, in the United States (US), exception from informed consent (EFIC) is used instead, governed by the Food and Drug Administration legislation. This is a term used to describe participant enrolment without prior consent, where the participant is informed at a later point in their recovery and consent may be sought. The US requires a public consultation exercise which assesses the suitability and community acceptability for each study before EFIC is granted. Differing provisions across the EU, US, and other jurisdictions make the use of deferred consent a complex but internationally relevant issue.

Set criterion must be fulfilled which permits its use in emergency settings or when the intervention is of minimal risk to the patient, for example where there is no alteration to the patient’s usual care [6]. The criterion for studies in EU member states is outlined in Table 1, although similar ethical requirements govern the use of deferred consent in other jurisdictions.

**Table 1:** Deferred consent criteria in Europe: existing laws and the EU Directive (adapted from Druml et al. [7] )

1.	The research is essential to validate data obtained in clinical trials on persons able to give informed consent or by other research methods
2.	The research has to relate directly to a life-threatening or debilitating clinical condition from which the incapacitated adult suffers
3.	The clinical trial has to be designed to minimise pain, discomfort, fear, and other foreseeable risks in relation to the disease and developmental stage
4.	The risk threshold and the degree of distress shall be specially defined and constantly monitored
5.	An ethics committee with expertise in the relevant disease and the patient population concerned or after taking advice in clinical, ethical, and psychosocial questions in the field of the relevant disease and patient population has endorsed the protocol
6.	The interests of the patient always prevail over those of science and society and the requirement that there are grounds for expecting that administering the medical product to be tested will produce a benefit to the patient that outweighs the risks or has no risk at all.
7.	Consider that scientifically unsound research is *ipso facto* unethical in that it may expose individuals to risks for no purpose at all.

Whilst this method offers the opportunity to enrol participants when a surrogate-decision maker is unavailable, it also may offer the potential to reduce selection bias. The requirement for all patients or their surrogates to provide informed consent before enrolment increases the potential for selection bias meaning research populations may not be representative of the typical patient [8] which can affect the ability to demonstrate the efficacy of the intervention [9]. Patients without a surrogate decision-maker have been shown to be characteristically different to those that do, and are often more critically unwell, further leading to the potential for an unrepresentative sample [10]. Therefore, whilst it is important to maintain clinical equipoise whilst conducting clinical trials with incapacitated patients, a balance must be achieved between conducting research ethically, whilst not discriminating against populations due to challenges or delays arising from the informed consent process. This highlights the need for other strategies, such as deferred consent, which has been found to increase the representation of patient minority groups in critical care studies [11]. However, there are ethical challenges in conducting research without prior informed consent, including the concern that it fails to meet researchers’ obligation to respect patients’ autonomy, which has led to empirical studies exploring stakeholders’ views about the acceptability of recruitment models such as deferred consent [12].

A growing awareness about the importance of representativeness of trial populations and the need for inclusivity in clinical trials is leading to a shift in focus on to *who* is included in research as opposed to *how many* [13]. Having an adequate sample size is important, but it is equally important to ensure that those included in a study adequately reflect the population for which the intervention is intended [13]. Evidence shows that less than a quarter of the global population are of European ancestry [14]. Therefore, studies that fail to diversify their patient samples lack generalisability and risk excluding groups with the highest disease burden [14]. Failure to adequately include minority groups in studies has contributed to treatment reluctancy amongst these under-served populations [13]. Initiatives such as the National Institute for Health Research (NIHR) INCLUDE project have identified a number of groups that are under-served in clinical research, for example those from low socioeconomic backgrounds, from ethnic minority groups, or with impaired capacity to consent, and have created a roadmap with suggested intervention points to improve inclusion [15]. However, the issue of inclusivity in methodological research, such as studies examining aspects of trial conduct like consent and recruitment, is one that needs addressing [15].

This commentary aims to explore ethnic and racial minority attitudes toward the use of deferred consent in adult clinical trials, and further consider its potential to improve trial inclusivity. We focus on ethnic minority groups specifically, with reference to other under-served populations identified by the INCLUDE project as appropriate. For the purposes of this exploration, exception from informed consent (EFIC) whereby consent may be gained after study enrolment as determined by US legislation, will also be considered.

## Main text

### Trial inclusivity

Patients recruited into clinical trials do not always reflect the composition of the general population, leading to research inequity. This means that trial outcomes may not reflect patient outcomes in clinical practice [16]. Evidence from the literature shows that many groups, for example, patients with a disability, are largely under-represented in clinical trials [17]. Reasons for this underrepresentation are due to multiple factors surrounding trial recruitment including a lack of understanding or confidence in recruiting patients from these groups [18]. For ethnic minorities, barriers to enrolment include healthcare mistrust and inadequate communication from healthcare professionals [19]. Themes of mistrust and communication are consistent across other underrepresented groups who are also excluded from research, such as patients with an intellectual disability [20]. Additionally, factors inherent to the study design, recruitment and consenting processes also contribute to underrepresentation such as stringent eligibility criteria, need for rapid recruitment and lack of research staff experience [21, 22].

COVID-19 research has thrown a spotlight on the issue of health disparity in treatment trials [23]. An analysis of trials of COVID-19 treatments in the US found that vulnerable populations, such as adults over 65 years, over 85 years and pregnant women were more likely to be excluded from clinical trials. In addition, trials were conducted in hospitals where there were less likely to be patients of Black and Hispanic ethnic origin [24]. This has raised important questions during the global pandemic, as to whether patients are adequately represented in clinical trials. These questions also extend to whether research exploring trial conduct, for example, surveys of attitudes towards recruitment methods, is inclusive. Ethically complex methods, like deferred consent, may be viewed differently by patients from under-served groups. This challenges the use of deferred consent in practice, particularly amongst these populations.

### Acceptability of deferred consent

Many studies have assessed attitudes toward deferred consent, which is deemed generally acceptable by patients and their surrogate decision-makers in emergency situations [25]. However, the studies from which these conclusions are drawn do not consider attitudes toward deferred consent from underserved groups. Findings from a minimal risk study in acute stroke showed that 91% of trial respondents were opposed to its use, where the sample was composed of a mixture of patients and their surrogates, 93% of whom were white. However, this failed to consider that patients and their surrogates may express different views. Another study that only assessed views from participants that regained capacity and did not include ethnicity data showed that 22% participants had a neutral or negative response as to whether the research team had made the right decision to defer consent [26]. The clarification between patient and surrogate views may be crucial, especially as patients that lack capacity are under-served, and often report different reasons for providing consent compared to their surrogate decision-makers [11, 27–29]. This consideration may partially explain the variation in acceptability seen across the literature. Additionally, the acceptability of deferred consent may be further confounded by the evaluation of its use when experienced first-hand, as opposed to through a hypothetical scenario, which has also been seen in minority sub-populations [30, 31].

### Attitudes of ethnic minorities towards deferred consent

Many of the terms used to describe different ethnicities and races within studies are subjective. Groups are recognised as more heterogenous than their descriptors imply but for the purposes of this article will be referred to as how they are described in the studies reviewed. A US survey conducted in the emergency setting exploring attitudes of exception from informed consent found that responses differed based on ethnicity. “Non-white” participants were less likely to agree that it may be appropriate to enrol participants without prior consent [32]. Although an important overall finding, the lack of distinction between “non-white” participants in the analysis may be significant. Closer inspection of the sample revealed that there were 110 (21%) African Americans, 9 Asian (1%), 29 Hispanic (6%) and 20 Native American (4%) participants. The grouping of these categories in the analysis considers these ethnicities to be more homogenous than they are. This is an important consideration, as historical mistrust has been shown to vary amongst ethnicity, generally with populations of African descent impacted the most [33, 34]. Another issue is the inclusion of 22 participants (3%) belonging to the “other or mixed” ethnic category within the sample size. This is problematic due to the ambiguous ethnic diversity within this group, which is likely to impact views on exception from informed consent. Additionally, “non-white” participants were more likely to disagree with a statement demonstrating willingness to participate in the study if it were important to learn about a treatment for a condition with no current beneficial treatment. In contrast, this group were more likely to agree that there are times when it is important to learn about a new treatment and in this case, it would be acceptable to enrol participants without prior consent. This shows that ethnic minorities are more likely to appreciate the importance of deferring consent but experience personal reluctancy toward enrolment regarding themselves. Furthermore, this demonstrates that the wording of statements and the language used when asking opinions of deferred consent may greatly impact the responses generated [35].

The Established Status Epilepticus Treatment Trial (ESETT) enrolled participants without prospectively provided consent and supported the finding that the phrasing of statements is important [35]. This is supported by studies showing acceptability varies when asked about personal or general enrolment using deferred consent [36]. In the ESETT trial, participants were asked if they were “glad that themselves or their family members were included in the study” [35]. There were no differences due to race on acceptance of enrolment. However, when asked if they thought that it was acceptable to include themselves or family members without asking for permission first, racial differences began to emerge. More black participants disagreed with enrolment without prior consent than white, other race and unknown race categories [35]. This finding may reflect other studies that show black participants prefer to give prior permission before their inclusion in research [37]. However, these findings may lead to numerous assumptions. Patient groups are categorised with terms such as “black” or “white” widely across the literature. This terminology fails to account for key inter-personal differences between participants and risks oversimplifying ethnically diverse groups of individuals.

As with some of the issues identified previously, an examination of studies investigating the impact of traumatic brain injury experience on exception from informed consent found that black participants were less likely to accept a situation with no prospectively provided consent. Additionally, those in the “other” category and those of Asian background had the lowest acceptance rates [30]. However, they also found that these differences were largely impacted by the previous first or second-hand experience of traumatic brain injury. There were similar levels of acceptance between ethnicities for those who had no personal traumatic brain injury experience. However, amongst white participants, the closer the connection with traumatic brain injury (i.e., a close family member), the greater the level of acceptance. In contrast, no increase was seen amongst black participants with a close connection with traumatic brain injury [30]. Therefore, experience may account for some of the differences seen amongst ethnicities and is supported by evidence from focus groups [38]. This finding is unsurprising, as experience is a strong predictor of acceptability towards the use of deferred consent amongst all key stakeholders involved [39, 40]. Additionally, those from ethnic minorities may have less personal experience of situations where deferring informed consent was necessary or may be more likely to have had adverse experiences of its use. This is supported by evidence showing that black participants are the largest racial group enrolled into studies using deferred consent, despite studies showing they express greater aversion to its use [30].

## Discussion

Whilst it is important that inclusivity in clinical trials is improved, how this should be addressed with respect to under-served groups such as ethnic minorities remains an issue. The need for inclusivity applies to COVID-19 research as well as trials in other conditions and has been described as a necessity rather than a luxury [13]. In emergency research, evidence regarding attitudes from under-served groups is important in order to establish whether deferred consent can aid inclusivity when used in practice. However, current studies fail to take account of the views of diverse ethnic groups about the complex ethical issues that arise in alternative consent models. Studies differentiate samples based on age and sex but often fail to report the ethnicity of patients included [41]. Even within those that do, participants are categorised in a way that shows greater genetic heterogeneity within than between racial or ethnic groups [14]. Often groups are perceived as more homogenous than they are, for example, “mixed-race” categories. Another issue is the representativity of the sample sizes. Many of the studies established conclusions regarding the whole ethnic minority population with only a few participants representative of that ethnicity. This defeats the purpose of diversifying research, as if the research regarding the way in which we conduct research is not representative, it is unlikely to help to diversify recruitment into clinical trials. Studies also fail to adjust for socioeconomic factors such as income level or years of education, which will ultimately impact life experiences [14], in addition to failing to acknowledge the intersectionality between different under-served groups. Failure to capture these differences may stigmatise and distort their conclusions.

This article has explored important differences for acceptability of deferred consent amongst under-served populations, showing that differences are impacted by prior experience, which may have a greater impact than ethnicity alone. Similarly, black participants placed greater value on giving permission for research. This may provide evidence that acceptability of deferred consent in patients is dependent upon personal experiences within the medical system, which may be crucial when superimposed on a background of historical mistrust [35]. This highlights that the interrelation of these factors requires further examination, and there is a need to recognise that recruitment must adapt to the relevant target population [32]. Exploring the information currently provided to participants and their surrogates when seeking consent to continue in a study, and understanding how this information is viewed by members of different ethnic minority groups, may also be helpful.

An important consideration is that studies assessing attitudes towards deferred consent often fail to distinguish views of patients and surrogates. For example, the finding that there is less acceptability from ethnic minority groups incorporates both surrogate-decision makers and previously incapacitated patients (an under-served group) with no distinction between them [42, 43]. The lack of clarity between these groups risks inaccurately over-representing minority views, further raising the issue of intersectionality whereby incapacitated patients are already likely to be under-served in deferred consent research. When these patients are from ethnic minority backgrounds then the issue of under-representation is synergised.

A fine balance must be achieved between adequately representing ethnic minorities in research and ensuring that participants are not over-enrolled due to deferred consent. Concerns have been raised about racial enrolment in emergency settings where in cases of severe trauma there may be over-enrolment of ethnic minorities. This is problematic as it could lead to inaccurate assumptions about the intervention for the general population regarding its relative success or failure, and we must ensure that enrolment of ethnic minorities is equitable [16].

It is also important to recognise that many of the studies explored aspects of exception from informed consent in the US as opposed to deferred consent as used in the UK and beyond. Whilst the two describe situations with no prospective prior consent, there is a difference regarding legislation surrounding their use. In EFIC, there may be no consent sought after participant enrolment. Attitudes have been shown to vary between the two with respect to cultural and regional differences [44]. However, many comparisons can be drawn between them, especially as the studies included in this article mainly consider acceptability surrounding enrolment without prior consent more broadly.

## Conclusions

Deferred consent undoubtedly improves trial recruitment numbers in emergency research and has not only been important in order to reach recruitment targets, but also to incorporate ethnic minorities where they may have been otherwise under-represented [45]. Overall findings reflect a combination of historical mistrust amongst vulnerable populations and the impact of prior experience [32]. Greater consideration is needed towards the categorisation of different ethnic groups. The small numbers of ethnic minorities within samples highlights the need for inclusivity within research on trial conduct, to avoid drawing incorrect conclusions and ensuring equitable opportunity for participation in trials. A balance must be achieved with regards to the enrolment of under-served populations to avoid potential over-enrolment in some circumstances. Additionally, understanding how certain ethnic groups value aspects of consent will aid equitable enrolment and increase trust amongst minority populations [45]. More effort is needed to build relationships between researchers and diverse populations and establish the drivers behind differences in attitudes toward recruitment methods like deferred consent. This will ultimately help to diversify those included in clinical trials, which is especially important in emergency and pandemic situations.

### Future implications

Previous work looking at how we can improve the conduct of trials has begun to consider the inclusivity of ethnic groups, such as the INCLUDE Ethnicity Framework hosted by Trial Forge [46]. Work is now needed to explore how we access and encourage under-served groups to participate in clinical trials. Examples of public engagement projects include “Talking trials” which has been set up by the Centre for Trials Research at Cardiff University, to conduct focus-groups with individuals from ethnic minority backgrounds to address ethical concerns surrounding participation in clinical trials [47]. Trial conduct research concerning trial participation should aim to better involve the public and ensure diverse recruitment that is reflective of the population. It is important to ensure that when we are considering the views of under-served groups, those involved are themselves representative at all stages and in all contributing roles. Findings from the INCLUDE project regarding COVID-19 research have supported this, suggesting that under-served groups should be adequately represented throughout. This includes diversifying the research process by adequately representing under-served groups not only in clinical trials, but in surveys regarding study participation, within research teams and funding bodies, and throughout research ethics committees [13].

## Data Availability

Not applicable

## Abbreviations

EU: European Union
EFIC: Exception from informed consent
REC: Research Ethics Committee
ESETT: The Established Status Epilepticus Treatment Trial

## References

[1] Armstrong S, Langlois A, Laparidou D, Dixon M, Appleton JP, Bath PM, Snooks H, Siriwardena AN (2017). Assessment of consent models as an ethical consideration in the conduct of prehospital ambulance randomised controlled clinical trials: a systematic review. BMC Medical Research Methodology..

[2] Raven-Gregg T, Wood F, Shepherd V. Effectiveness of participant recruitment strategies for critical care trials: A systematic review and narrative synthesis. Clinical Trials. 2021;1740774520988678. 10.1177/1740774520988678 33530728

[3] Van der Graaf R, Hoogerwerf M-A, de Vries MC (2020). The ethics of deferred consent in times of pandemics. Nature Medicine..

[4] Flanagan BM, Philpott S, Strosberg MA (2011). Protecting Participants of Clinical Trials Conducted in the Intensive Care Unit. Journal of Intensive Care Medicine (Sage Publications Inc)..

[5] Harvey SE, Elbourne D, Ashcroft J, Jones CM, Rowan K (2006). Informed consent in clinical trials in critical care: experience from the PAC-Man Study. Intensive Care Medicine..

[6] Bigatello LM, George E, Hurford WE (2003). Ethical considerations for research in critically ill patients. Critical Care Medicine : official journal of the society of critical care medicine..

[7] Druml C (2004). Informed consent of incapable (ICU) patients in Europe: existing laws and the EU Directive. Current Opinion in Critical Care..

[8] Jansen TC, Kompanje EJ, Bakker J. Deferred proxy consent in emergency critical care research: ethically valid and practically feasible. Critical Care Medicine. 2009;37:S65-S68, Supplement, doi: 10.1097/CCM.0b013e3181920851. 10.1097/CCM.0b013e3181920851 19104227

[9] Holcomb JB, Weiskopf R, Champion H, Gould SA, Sauer RM, Brasel K, et al. Challenges to Effective Research in Acute Trauma Resuscitation: Consent and Endpoints. Shock. 2011;35(2). 10.1097/SHK.0b013e3181f7fd01 20926987

[10] Larkin ME, Beauharnais CC, Magyar K, Macey L, Grennan KB, Boykin EE, Russell SJ (2012). Obtaining surrogate consent for a minimal-risk research study in the intensive care unit setting. Clinical Trials..

[11] Burns KEA, Zubrinich C, Tan W, Raptis S, Xiong W, Smith O, McDonald E, Marshall JC, Saginur R, Heslegrave R, Rubenfeld G, Cook DJ, for the Canadian Critical Care Trials Group (2013). Research Recruitment Practices and Critically Ill Patients. A Multicenter, Cross-Sectional Study (The Consent Study). American Journal of Respiratory and Critical Care Medicine..

[12] Burkard J. The Importance of Using Deferred Proxy Consent in Emergency Clinical Research. Voices in Bioethics. 2018;4, 4.

[13] Witham MD, Anderson E, Carroll C, Dark PM, Down K, Hall AS, Knee J, Maier RH, Mountain GA, Nestor G, Oliva L, Prowse SR, Tortice A, Wason J, Rochester L, On behalf of the INCLUDE writing group (2020). Developing a roadmap to improve trial delivery for under-served groups: results from a UK multi-stakeholder process. Trials..

[14] Wilkins CH, Schindler SE, Morris JC (2020). Addressing Health Disparities Among Minority Populations: Why Clinical Trial Recruitment Is Not Enough. JAMA Neurology..

[15] National Institute for Health Research. Improving inclusion of under-served groups in clinical research: Guidance from INCLUDE project. 2020 07/08/2020.

[16] Sugarman J, Sitlani C, Andrusiek D, Aufderheide T, Bulger EM, Davis DP, Hoyt DB, Idris A, Kerby JD, Powell J, Schmidt T, Slutsky AS, Sopko G, Stephens S, Williams C, Nichol G, Resuscitation Outcomes Consortium Investigators (2009). Is the enrollment of racial and ethnic minorities in research in the emergency setting equitable?. Resuscitation..

[17] Feldman MA, Bosett J, Collet C, Burnham-Riosa P (2014). Where are persons with intellectual disabilities in medical research? A survey of published clinical trials. Journal of Intellectual Disability Research..

[18] Shepherd V, Griffith R, Sheehan M, Wood F, Hood K (2018). Healthcare professionals' understanding of the legislation governing research involving adults lacking mental capacity in England and Wales: a national survey. Journal of Medical Ethics..

[19] Hamel LM, Penner LA, Albrecht TL, Heath E, Gwede CK, Eggly S (2016). Barriers to Clinical Trial Enrollment in Racial and Ethnic Minority Patients With Cancer. Cancer Control : Journal of the Moffitt Cancer Center..

[20] Shepherd V, Wood F, Griffith R, Sheehan M, Hood K (2019). Protection by exclusion? The (lack of) inclusion of adults who lack capacity to consent to research in clinical trials in the UK. Trials..

[21] Fletcher JR. Unethical governance: capacity legislation and the exclusion of people diagnosed with dementias from research. Research Ethics. 2020;1747016120982023.

[22] Trivedi RB, Humphreys K (2015). Participant exclusion criteria in treatment research on neurological disorders: Are unrepresentative study samples problematic?. Contemporary Clinical Trials..

[23] Flores LE, Frontera WR, Andrasik MP, del Rio C, Mondríguez-González A, Price SA (2021). Assessment of the Inclusion of Racial/Ethnic Minority, Female, and Older Individuals in Vaccine Clinical Trials. JAMA Network Open.

[24] Chokkara S, Volerman A, Ramesh S, Laiteerapong N (2021). Examining the Inclusivity of US Trials of COVID-19 Treatment. Journal of General Internal Medicine..

[25] Kompanje EJO, van Dijck JTJM, Chalos V, van den Berg SA, Janssen PM, Nederkoorn PJ, van der Jagt M, Citerio G, Stocchetti N, Dippel DWJ, Peul WC (2020). Informed consent procedures for emergency interventional research in patients with traumatic brain injury and ischaemic stroke. The Lancet Neurology..

[26] Terry MA, Freedberg DE, Morris MC (2017). An Alternative Consent Process for Minimal Risk Research in the ICU. Critical Care Medicine..

[27] Bolmsjo I, Hermeren G (2001). Interviews with patients, family, and caregivers in amyotrophic lateral sclerosis: comparing needs. Journal of Palliative Care..

[28] Coppolino M, Ackerson L (2001). Do surrogate decision makers provide accurate consent for intensive care research?. Chest..

[29] Upadya A, Muralidharan V, Thorevska N, Amoateng-Adjepong Y, Manthous CA (2002). Patient, Physician, and Family Member Understanding of Living Wills. American Journal of Respiratory and Critical Care Medicine..

[30] Scicluna VM, Ali MK, Pentz RD, Wright DW, Dickert NW (2017). Does experience matter? Implications for community consultation for research in emergency settings. AJOB Empirical Bioethics..

[31] Scales DC, Smith OM, Pinto R, Barrett KA, Friedrich JO, Lazar NM, Cook DJ, Ferguson ND (2009). Patients' preferences for enrolment into critical-care trials. Intensive Care Medicine..

[32] McClure KB, DeIorio NM, Gunnels MD, Ochsner MJ, Biros MH, Schmidt TA (2003). Attitudes of Emergency Department Patients and Visitors Regarding Emergency Exception from Informed Consent in Resuscitation Research, Community Consultation, and Public Notification. Academic Emergency Medicine..

[33] Kennedy BR, Mathis CC, Woods AK (2007). African Americans and their distrust of the health care system: healthcare for diverse populations. Journal of Cultural Diversity..

[34] Scharff DP, Mathews KJ, Jackson P, Hoffsuemmer J, Martin E, Edwards D (2010). More than Tuskegee: understanding mistrust about research participation. Journal of Health Care for the Poor and Underserved..

[35] Scicluna VM, Biros M, Harney DK, Jones EB, Mitchell AR, Pentz RD, Silbergleit R, Speight CD, Wright DW, Dickert NW (2020). Patient and Surrogate Postenrollment Perspectives on Research Using the Exception From Informed Consent: An Integrated Survey. Annals of Emergency Medicine..

[36] Feldman WB, Hey SP, Franklin JM, Kesselheim AS (2019). Public Approval of Exception From Informed Consent in Emergency Clinical Trials: A Systematic Review of Community Consultation Surveys. JAMA Network Open.

[37] Hull SC, Sharp RR, Botkin JR, Brown M, Hughes M, Sugarman J, Schwinn D, Sankar P, Bolcic-Jankovic D, Clarridge BR, Wilfond BS (2008). Patients' Views on Identifiability of Samples and Informed Consent for Genetic Research. The American Journal of Bioethics..

[38] Richardson LD, Wilets I, Ragin DF, Holohan J, Smirnoff M, Rhodes R (2005). Research without Consent: Community Perspectives from the Community VOICES Study. Academic Emergency Medicine..

[39] Williams MA, Haywood C (2003). Critical care research on patients with advance directives or do-not-resuscitate status: ethical challenges for clinician-investigators. Critical Care Medicine : official journal of the society of critical care medicine..

[40] Gobat NH, Gal M, Francis NA, Hood K, Watkins A, Turner J, Moore R, Webb SAR, Butler CC, Nichol A (2015). Key stakeholder perceptions about consent to participate in acute illness research: a rapid, systematic review to inform epi/pandemic research preparedness. Trials..

[41] Flanagin A, Frey T, Christiansen SL, Bauchner H (2021). The Reporting of Race and Ethnicity in Medical and Science Journals: Comments Invited. JAMA..

[42] Dickert NW, Mah VA, Baren JM, Biros MH, Govindarajan P, Pancioli A, Silbergleit R, Wright DW, Pentz RD (2013). Enrollment in research under exception from informed consent: the Patients' Experiences in Emergency Research (PEER) study. Resuscitation..

[43] Dickert NW, Scicluna VM, Baren JM, Biros MH, Fleischman RJ, Govindarajan PR, Jones EB, Pancioli AM, Wright DW, Pentz RD (2015). Patients' perspectives of enrollment in research without consent: the patients' experiences in emergency research-progesterone for the treatment of traumatic brain injury study. Critical Care Medicine..

[44] Grap MJ, Munro CL (2003). Subject recruitment in critical care nursing research: a complex task in a complex environment. Heart & Lung..

[45] Clifton GL, Knudson P, McDonald M (2002). Waiver of Consent in Studies of Acute Brain Injury. Journal of Neurotrauma..

[46] Trial Forge. Trial Forge - The INCLUDE Ethnicity Framework 2020 [Available from: https://www.trialforge.org/trial-forge-centre/include/.

[47] Centre for Trials Research (2020). Talking Trials - Centre for Trials Research - Cardiff University.

